# The complete mitochondrial genome of *Cynops cyanurus cyanurus* (Caudata: Salamandridae) and its phylogenetics analysis

**DOI:** 10.1080/23802359.2020.1715887

**Published:** 2020-01-24

**Authors:** Hao Li, Lin Cui, Diyan Li, Xiaolan Fan, Mingyao Yang, Deying Yang, Qingyong Ni, Yan Li, Yongfang Yao, Huailiang Xu, Bo Zeng, Ying Li, Feida Sun, Mingwang Zhang

**Affiliations:** a College of Animal Science and Technology, Sichuan Agricultural University, Chengdu, P. R. China;; b Animal Genetic Resources Exploration and Innovation Key Laboratory of Sichuan Province, Sichuan Agricultural University, Chengdu, Sichuan, China;; c College of Life Science, Sichuan Agricultural University, Sichuan, P. R. China

**Keywords:** *Cynops cyanurus* cyanurus, complete mitogenome, Salamandridae

## Abstract

The complete mitochondrial of *Cynops cyanurus cyanurus* was determined by using Sanger sequencing and the mitogenome size of *C. c. cyanurus* was 16,465 bp. It contained 13 protein-coding genes, 22 tRNAs, 2 rRNAs, 1 control region (D-loop) and a non-coding region (NC). In addition, the phylogenetic tree shows that *C. c. cyanurus* has the closest relationship with *C. c. chuxiongensis*. Hoping this study can improve our understanding of the species evolutionary relationship of *Cynops.*

Mainly distributed in the southwest of China, the species *Cynops cyanurus* is composed of two subspecies: *C. c. chuxiongensis* and *C. c. cyanurus.* The adult newts hibernate from October to March of the following year, mostly in damp caves or caves near the water, and prey mainly on small animals such as aquatic insects, slugs, and water fleas in the water (Fei and Ye 1983). A recent study has reported the subspecies mitogenome of *C. c. chuxiongensis* (Cui et al. 2020), but not that of *C. c. cyanurus*. The current study explores the mitogenome of *C. c. cyanurus* and assessed phylogenetic relationships among salamanders of the family Salamandridae.

The sample of *C. c. cyanurus* was collected in Wenshan autonomous prefecture (23°41′31.38″N 105°9′55.18″E), Yunnan province, China, at an altitude of 1806 meters. Tail tip tissue was stored in absolute ethanol at −80 °C (Accession number: LJ20). The genomic DNA was extracted using Ezup-pillar Genomic DNA Extraction kit (Sangon, Shanghai, China), followed the manufacturer’s instructions. Methods for DNA amplification and sequencing were performed as in Cui et al. (2020). The new complete mitogenome of *C. c. cyanurus* was annotated using the MITOS web server under the genetic code for vertebrates (Matthias et al. 2013) and then submitted to GenBank with an accession number MF037878.

The circular complete mitogenome of *C. c. cyanurus* is 16,465 bp in length. The base composition is 32.5% A, 28.1% T, 24.0% C, and 15.4% G. The mitogenome contains 37 genes, including 13 protein-coding genes (PCGs), 22 transfer RNA (tRNA), 2 ribosomal RNA (rRNA), one control region (D-loop) and one non-coding region (NC). The two rRNA genes (12 s rRNA, 16 s rRNA) were 929 bp and 1558 bp long, respectively. All protein-coding genes initiate with an ATG start codon, except for COXI, which uses GTG as the start codon. The protein-coding genes have three different types of stop codons. ND1 and COXI use TAG as their stop codons; ND6 has a stop codon with AGA; and the remaining genes use TAA as their stop codons. The control region (D-loop) is 732 bp in length and one non-coding (NC) region (327 bp) is found between tRNA^Thr^ and tRNA^Pro^. Most of the genes were encoded on the heavy strand. Only ND6 and other eight tRNA genes (tRNA^Gln^, tRNA^Ala^, tRNA^Asn^, tRNA^Cys^, tRNA^Tyr^, tRNA^Ser(UCN)^, tRNA^Glu^, and tRNA^Pro^) were encoded on the light strand.

In order to evaluate the relationship of the two subspecies and other salamanders of the family Salamandridae, the phylogenetic tree was constructed using MrBayes method by PhyloSuite (Zhang et al. 2020), which is based on the concatenated 13 PCGs of 22 species from seven genera (*Tylototriton, Triturus, Cynops, Laotriton, Pachytriton, Paramesotriton*, and *Alligatorinae*). *Alligator mississippiensis* was used as an outgroup. The result suggests that *C. c. cyanurus* has the closest relationship with *C. c. chuxiongensis* (Figure 1) and our results of phylogenetic relationships of the salamander family Salamandridae also agree with the previous study reported by Zhang et al. (2008). The new mitogenome sequence in this study will contribute to the classification of *Cynops* and the protection of genetic resources.

**Figure 1.**
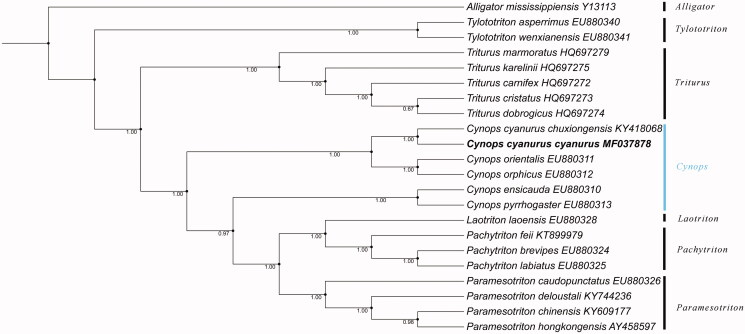
Mr. Bayes phylogenetic tree of salamanders based on 13 protein-coding genes. The numbers at each node indicate the Bayesian posterior probabilities. The GenBank accession numbers are listed following species names.
